# R54C Mutation of *NOTCH3* Gene in the First Rungus Family with CADASIL

**DOI:** 10.1371/journal.pone.0135470

**Published:** 2015-08-13

**Authors:** Kheng-Seang Lim, Ai-Huey Tan, Chun-Shen Lim, Kek-Heng Chua, Ping-Chin Lee, Norlisah Ramli, Giri Shan Rajahram, Fatimah Tina Hussin, Kum-Thong Wong, Meenakshi B. Bhattacharjee, Ching-Ching Ng

**Affiliations:** 1 Division of Neurology, Faculty of Medicine, University of Malaya, Kuala Lumpur, Malaysia; 2 Genetics and Molecular Biology Unit, Institute of Biological Sciences, Faculty of Science, University of Malaya, Kuala Lumpur, Malaysia; 3 Department of Biomedical Science, Faculty of Medicine, University of Malaya, Kuala Lumpur, Malaysia; 4 Department of Biomedical Imaging, Faculty of Medicine, University of Malaya, Kuala Lumpur, Malaysia; 5 Department of Medicine, Hospital Queen Elizabeth, Sabah, Malaysia; 6 Department of Radiology, Hospital Queen Elizabeth, Sabah, Malaysia; 7 Department of Pathology, Faculty of Medicine, University of Malaya, Kuala Lumpur, Malaysia; 8 Department of Pathology and Laboratory Medicine, UT Health Science Center, Houston, TX, United States of America; Nathan Kline Institute and New York University School of Medicine, UNITED STATES

## Abstract

Cerebral autosomal dominant arteriopathy with subcortical infarcts and leukoencephalopathy (CADASIL) is a rare hereditary stroke caused by mutations in *NOTCH3* gene. We report the first case of CADASIL in an indigenous Rungus (Kadazan-Dusun) family in Kudat, Sabah, Malaysia confirmed by a R54C (c.160C>T, p.Arg54Cys) mutation in the *NOTCH3*. This mutation was previously reported in a Caucasian and two Korean cases of CADASIL. We recruited two generations of the affected Rungus family (*n* = 9) and found a missense mutation (c.160C>T) in exon 2 of *NOTCH3* in three siblings. Two of the three siblings had severe white matter abnormalities in their brain MRI (Scheltens score 33 and 50 respectively), one of whom had a young stroke at the age of 38. The remaining sibling, however, did not show any clinical features of CADASIL and had only minimal changes in her brain MRI (Scheltens score 17). This further emphasized the phenotype variability among family members with the same mutation in CADASIL. This is the first reported family with CADASIL in Rungus subtribe of Kadazan-Dusun ethnicity with a known mutation at exon 2 of *NOTCH3*. The penetrance of this mutation was not complete during the course of this study.

## Introduction

Cerebral autosomal dominant arteriopathy with subcortical infarcts and leukoencephalopathy (CADASIL) was characterized by migraine, recurrent subcortical stroke and dementia. CADASIL is caused by a genetic mutation in the notch homolog protein 3 (*NOTCH3)* gene [1], that encodes a transmembrane receptor located on the surface of the arterial smooth muscles, with (i) an extracellular domain consisting of 34 epidermal growth factor-like repeats (EGFRs) and three Notch/Lin12 repeats, (ii) a transmembrane domain, and (iii) an intracellular domain consisting of seven ankyrin repeats. It was recently shown that aggregation/accumulation of NOTCH3 with mutations in the extracellular domains (Notch3^ECD^) in the brain vessels is a central event, promoting the abnormal recruitment of functionally important extracellular matrix proteins that may ultimately cause multifactorial toxicity [2]. This disorder, which was reported initially in European Caucasian families [3, 4], is increasingly being recognized among the Asian [5–7].

*NOTCH3* mutations among individuals with CADASIL are mostly located in exon 4, followed by exons 3, 5, 6, and 11 [8–11]. Till 2009, more than 170 mutations were reported in people of many ethnic origins [12], and yet new mutations are still being discovered since then, especially in ethnicity not previously reported [13–16]. In Malaysia, only a Chinese family with eight members having typical clinical features and genetic confirmation of CADASIL was described in 2004 [17], but not in other ethnicity.

Here we report a familial CADASIL in a Rungus family, an indigenous tribe that has not been reported previously. We found R54C (c.160C>T) mutation of *NOTCH3* gene in two clinically affected members and one asymptomatic carrier. Rungus belongs to a subtribe of Kadazan-Dusun residing in Kudat, Sabah (North Borneo), Malaysia.

## Methods and Patients

**Fig 1:**
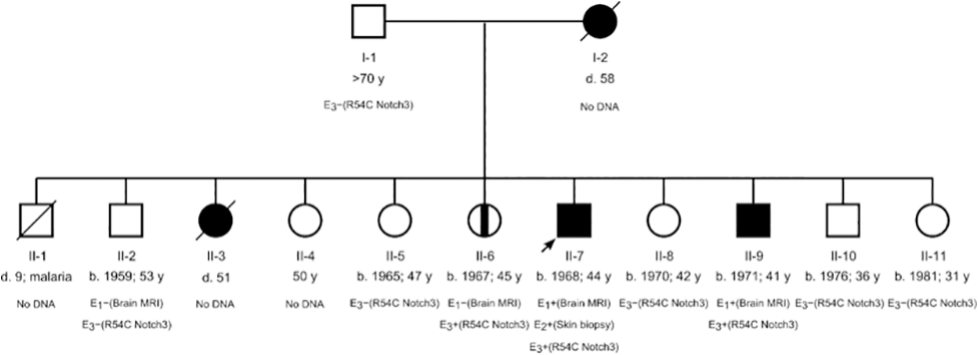
Pedigree for two generations of the Rungus family affected by CADASIL. Square and circle denote male and female, respectively. Solid square and circle denote affected male and female, respectively. Vertical band (II-6) denotes asymptomatic mutation carrier. The proband is denoted with an arrow. A strikethrough denotes a decreased member. E_1_, E_2_ and E_3_ denote brain MRI, skin biopsy and genetic evaluation, respectively.

The proband (II-7; Fig 1) with migraine and strong family history of young stroke was identified and confirmed to have CADASIL based on typical white matter changes in the MRI brain and electron microscopic abnormalities of the skin biopsy.

### Recruitment of families with CADASIL and clinical assessment

Ethical approval was obtained from the ethics committee at the University Malaya Medical Centre and Medical Review & Ethics Committee, Ministry of Health, Malaysia for assessment of the family of the index case. Written consents were obtained. A detailed clinical assessment of the family members was performed with regards to the presence of stroke, migraine and dementia, using semi-structured interview and structured questionnaires. In addition, new presentations that were not described before were explored. A detailed pedigree was plotted.

### Neuroimaging study

Three family members (II-6, II-7 and II-9) underwent magnetic resonance imaging (MRI) of the brain using a 1.5T Avanto (Siemens, Erlangen, Germany). The MRI sequences utilized were Axial T2W, T1W, fluid attenuation inversion recovery (FLAIR) and magnetic resonance angiography (MRA). Neuroradiologist, blinded to the clinical details of the subjects, graded the images. The lesions are quantified by Scheltens scoring system [18] and lesion distribution assessment (LDA) as described by Coulthard et al [19].

### Blood samples collection, DNA extraction and mutations screening

About 3 ml of peripheral blood was collected from each member. Genomic DNA was extracted using QIAamp DNA Blood Midi kit (Qiagen, Hilden, Germany) according to the manufacturer's instructions. The twenty-three exons of *NOTCH3* of the proband were amplified by PCR using Q5 hot start high-fidelity DNA polymerase (NEB, Ipswich, MA, USA) or Phusion Flash high-fidelity PCR master mix (Finnzymes, Thermo Scientific, Lafayette, CA, USA) (see supplementary materials). The PCR products were purified followed by DNA sequencing. The sequencing results were compared with *NOTCH3* refseq (GenBank: NG_009819). We identified two missense mutations, c.160C>T (R54C) in exon 2 and c.1490C>T (rs114207045; S497L) in exon 9 of *NOTCH3*. Functional impact of these two mutations were annotated using Polyphen-2 v2.2.2 (Polymorphism Phenotyping v2) prediction scores.[20] An amino acid change is predicted as probably damaging if its probability score is greater than 0.85 or as “possibly damaging” if the score is between 0.85 and 0.15 and benign for the remaining. Sanger sequencing was then performed to determine the presence of c.160C>T and c.1490C>T *NOTCH3* mutations in other family members and subsequently c.160C>T *NOTCH3* mutation in100 normal Rungus individuals.

## Results

### Case Description

#### Family pedigree

Out of 13 identifiable family members extending over 2 generations, four (2 deceased: I-2 and II-3; Fig 1) were clinically affected. Two symptomatic (II-7 and II-9) and one asymptomatic subject (II-6) were genetically confirmed. Main clinical features observed in this family include migraine, young stroke (before the age of 50), neuropsychiatry symptoms presenting as gelastic episodes, and dementia. Table 1 summarizes their clinical presentations, disease progression and MRI findings. Two demonstrative cases, the index case (II-7) and his younger brother (II-9) are fully reported here.

**Table 1:** Demographics and clinical presentations of affected family members.

Family member/Sex	Age of onset	Age at assessment	Vascular risk factors	Clinical features and progression of illness
I-2/F	40	NA	None	40y: Migraine
				48y: Gelastic episodes
				49y: Hemiparetic stroke followed by progressive motor decline
				53y: Dementia
				58y: Passed away
II-3/F	43	NA	None	43y: Migraine
				48y: Hemiparetic stroke, followed by gelastic episodes and dementia
				51y: Passed away
II-6/F	NA	45	None	No history of migraine and stroke
II-7/M	42	44	Dyslipidemia	42y: Migraine
				45y: Noted to have mild cognitive impairment
II-9/M	38	41	None	38y: Hemiparetic stroke followed by gelastic episodes and dementia

NA, not applicable

#### Case II-7 (Proband)

**Fig 2:**
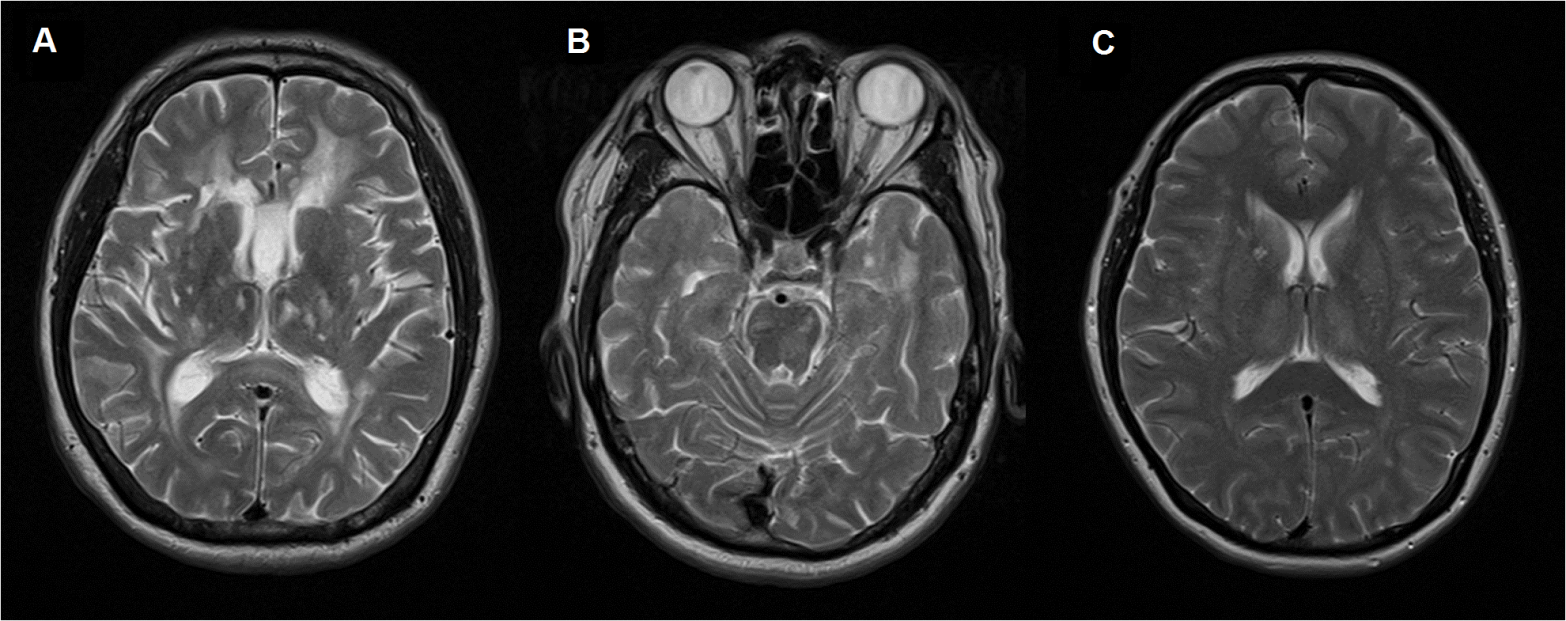
Electron microscopy of skin biopsy shows electron dense granular deposits (arrows) in the basal lamina surrounding vascular smooth muscle cells.

This 45 years old school teacher presented with recurrent episodes of migraine since 42 years old. The headache was left sided and preceded by visual aura, occurred daily and lasting one hour, responded well to amitriptyline. Neurological examination was normal. He scored 22/30 on Montreal Cognitive Assessment (MOCA). Trail making, Rey-Osterrieth figure drawing and Neuropsychiatry Inventory (NPI) tests were normal. Electron microscopic examination of the skin biopsy showed granular osmophilic material (GOM) adjacent to vascular smooth muscle cells. (Fig 2)

#### Case II-9

This 40 years old man farmer presented with sudden onset right hemiparesis at the age of 38. Subsequently, he developed progressive dementia and had gelastic episodes, where he was frequently described to be laughing inappropriately in many circumstances. He also had marked difficulty in falling asleep at night. He denied any history of migraine. On examination, he was able to obey simple command. The powers of the right upper and lower limb were 3/5. MOCA score was only 9/30 and he could not complete the trail making and Rey-Osterrieth figure drawing tests. In addition, NPI assessment revealed mild depression and anxiety intermixed with episodes of euphoria, which did not affect his work routine.

#### Neuroimaging findings

**Fig 3:**
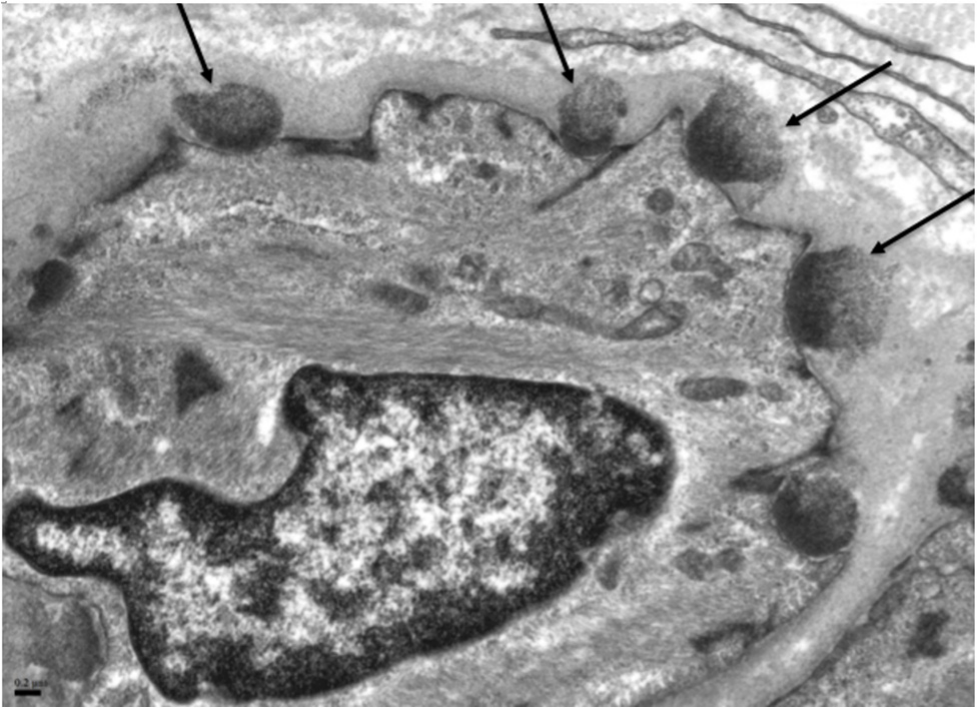
(A): Axial T2W MRI brain of a symptomatic subject (II-9) demonstrates cerebral atrophy. There are confluent hyperintense lesions in the periventricular, subcortical, deep white matter and thalamus, with (B) infra-tentorial and anterior temporal lobe involvement. 3(C): Axial T2W MRI brain of an asymptomatic subject (II-6) demonstrates more discreet deep white matter and periventricular lesions.

The Scheltens score were higher (33 and 50 respectively) for the 2 symptomatic subjects (II-7 and II-9) than the asymptomatic subject (II-6), whose score was 17 (Table 2). All the subjects demonstrated lesions in the periventricular and white matter regions of the frontal and parietal lobes. The symptomatic subject with the highest score (II-9) had lesions affecting the thalamus and infra-tentorial region (Fig 3A and 3B), and more numerous and larger white matter lesions, as compared with the asymptomatic subject demonstrating discreet lesions (Fig 3C). We also recorded anterior temporal and external capsule white matter changes in the symptomatic subjects (Fig 3B). The LDA scores follow similar trends to the Scheltens score (Table 2). Atrophy is more marked in the subjects with the higher Scheltens and LDA score. All the subjects demonstrated normal MR angiographic findings with no evidence of intracranial vascular stenosis seen.

**Table 2:** Scheltens scores and lesion distribution assessment (LDA) scores of the CADASIL family members.

	II-6	II-7	II-9
Scheltens scores			
Periventricular hyperintensities^1^ (PVH 0–6)			
Capsular occipital	1	2	2
Capsular frontal	1	2	2
Bands lateral ventricle	1	2	2
White matter hyperintensities^2^ (WMH 0–24)			
Frontal	4	6	6
Parietal	4	6	6
Temporal	0	6	6
Occipital	0	0	6
Basal ganglia hyperintensities^2^ (BG 0–30)			
Caudate	0	3	0
Putamen	0	3	0
GP	0	0	0
Thalamus	0	0	4
Internal capsule	3	3	3
Infratentorial foci of hyperintensity^2^ (ITF 0–24)			
Cerebellum	0	0	3
Midbrain	0	0	3
Pons	3	0	4
Medulla	0	0	3
**Total**	**17**	**33**	**50**
LDA scores^3^			
Periventricular			
Frontal	1	3	3
Parietal	1	3	3
Temporal	1	3	3
Occipital	1	3	3
Subcortical			
Frontal	3	3	3
Parietal	3	3	3
Temporal	1	3	3
Occipital	0	0	3
Deep grey matter			
Caudate	0	1	0
Putamen	0	1	0
GP	0	0	0
Thalamus	0	0	3
Brainstem	1	0	3
Cerebellum	0	0	1
Corpus callosum/splenium	0	0	2
**Total**	**12**	**23**	**33**
Other findings^3^			
Projection fibers (Internal capsule posterior limb)	0	1	2
Temporal white matter			
Anterior to posterior margin of amygdala	0	3	3
Posterior to posterior margin of amygdala	0	1	1
External capsule	0	3	3
Atrophy^4^	0	1	2

^1^ 0 = absent; 1 = 0 to 5 mm; 2 = > 5 mm

^2^ 0 = absent; 1 = up to five lesions of <3mm diameter; 2 = six or more lesions of <3mm; 3 = up to five lesions 4 to 10 mm in diameter; 4 = six or more lesions of 4 to 10mm; 5 = one or more lesions 10mm in size; and 6 = confluent hyperintensity

^3^ 0 = absent, 1 = <5 lesions, 2 = 5–10 lesions, 3 = >10 lesions

^4^ 0 = absent, 1 = mild, 2 = moderate, 3 = severe (sulcal, cerebellar folia prominence, enlargement of ventricles, brainstem size)

#### *NOTCH3* mutation

We sequenced exons 2 to 24 of *NOTCH3* of the proband. Exons 2–24 encode 34 EGF-like repeat domains of NOTCH3. Two heterozygous missense mutations, c.160C>T (R54C) in exon 2 and c.1490C>T (rs114207045; S497L) in exon 9 were identified. Polyphen-2 predicted R54C as a probably damaging variant (1.000) and S49L as a benign substitution (0.065). We therefore recruited two generations (*n* = 9) of the affected Rungus family for clinical and genetic evaluations (Fig 1; Table 1). We found that two of the proband's siblings (II-6 and II-9) have the exon 2 mutation, in which II-9 had young stroke and dementia, whereas II-6 was asymptomatic. We further sequenced exons 3–24 of *NOTCH3* of the two siblings. However, no additional missense mutations were found. Of the recruited members, I-1, II-2, II-5, II-7, II-8, and II-11 are the exon 9 mutation carriers but asymptomatic. In addition, we screened exon 2 of *NOTCH3* in 100 Rungus control individuals. None of the controls has the exon 2 mutation.

## Discussion

Despite over 600 families affected by CADASIL previously reported worldwide, to our knowledge, this is the first report of CADASIL in an indigenous tribe–Rungus, a Kadazan-Dusun subtribe diagnosed clinically, and confirmed genetically found to have a missense mutation, c.160C>T (R54C) in exon 2 of *NOTCH3* gene. Kadazan-Dusun is an indigenous tribe with a population of 568,000, residing mostly in Sabah in Borneo, the third largest island in the world and the largest island in Asia.

R54C mutation in exon 2 of *NOTCH3* gene is likely the pathogenic mutation in this family, which was previously reported in a Caucasian and two Korean cases as a causative mutation for CADASIL [21, 22] Functionally, the R54C mutation causes addition of a cysteine residue to the first EGFR of *NOTCH3*, resulting in anomalous pairings between seven cysteine residues during the formation of three disulfide bonds. The other missense mutation, i.e., c.1490C>T (rs114207045; S497L) in exon 9 identified in the proband is likely non-pathogenic, because (a) it is absent in case II-9 with strong clinical features of CADASIL, and (b) present in 5 members who were asymptomatic and likely unaffected. The exon 9 mutation is also a splice site mutation with a minor allele frequency of < 1% in Asian population as reported by 1000 Genomes Project [23]. Although protein modeling suggests that the exon 9 mutation S497L causes significant changes of *NOTCH3* secondary structure [24], genetic and clinical evidences suggesting association between the exon 9 mutation and cerebral small vessel disease/ischemic stroke are still lacking [25–28].

### Clinical variation

There was marked clinical variation in this family. Case II-6 was asymptomatic with the least white matter changes in the MRI despite being the oldest of the three cases. Both deceased cases (I-2 and II-3) were female with later stroke onset (49 and 48 years old, respectively) than case II-9 (38 years old). In a study of 411 patients in Germany, the clinical course of CADASIL was found to be variable [29]. Female was reported to have later onset of stroke and longer life expectancy than male, after adjusted for gender variation in normal population [29]. In addition, female was also found to have less lacunar infarcts compared with male [30]. Hypertension and smoking were associated with increased risk of stroke [31], but no difference in cardiovascular risk factors was found between the three cases in this family.

Despite carrying the exon 2 mutation, case II-6 did not show any clinical features of CADASIL with only minimal MRI changes as evidence by the lower Scheltens and LDA scores. These suggest that the penetrance rate might not be 100% among the mutation carriers. CADASIL was reported to be a disease with high penetrance, in which the penetrance in a large France family was reported to be complete after 20 years based on MRI changes [32]. In a 5-generational family in China, five members were reported as carrier but all had long T1 and T2 signals within the temporal lobes and high-intensity signals of FLAIR sequence [33].

The proband (II-7) had an extensive MRI changes but only presented with migraine, whereas case II-9 had developed stroke but without having a history of migraine. This difference in phenotype is related to the extent of white matter changes in the MRI brain. A history of migraine with aura might be protective against stroke as previously reported in those with migraine was associated with a reduced risk of stroke [31]. It is also postulated that having additional *NOTCH3* mutation in exon 9 in the proband might be protective against stroke by modifying the *NOTCH3* structure; however, this postulation requires further investigations.

## Conclusion

This is the first reported family with CADASIL in a Rungus subtribe of Kadazan-Dusun ethnicity, which carries a known *NOTCH3* mutation in exon 2, c.160C>T (R54C). The penetrance of this mutation was incomplete at the time of this report. MRI brain abnormality is a potential surrogate marker for early diagnosis, which provides a time window for potential primary stroke prevention.

## Supporting Information

S1 Table Primers for PCR and DNA sequencing. (DOCX)

S2 Table PCR working reaction for exon 2 and 24. (DOCX)

S3 Table PCR working reaction for exons 3–6, 7–10, 11–12, 13–16, 17–21, and 22–23. (DOCX)

S4 Table PCR cycling conditions. (DOCX)
