# Correction: R54C Mutation of *NOTCH3* Gene in the First Rungus Family with CADASIL

**DOI:** 10.1371/journal.pone.0138600

**Published:** 2015-09-14

**Authors:** Kheng-Seang Lim, Ai-Huey Tan, Chun-Shen Lim, Kek-Heng Chua, Ping-Chin Lee, Norlisah Ramli, Giri Shan Rajahram, Fatimah Tina Hussin, Kum-Thong Wong, Meenakshi B. Bhattacharjee, Ching-Ching Ng

The images for figures Figs 2 and 3 are swapped. The image for Fig 2 should be Fig 3 and the image for Fig 3 should be Fig 2. The figure captions are correct. The publisher apologizes for the error. Please see the corrected Figs 2 and 3 here.

**Fig 2:**
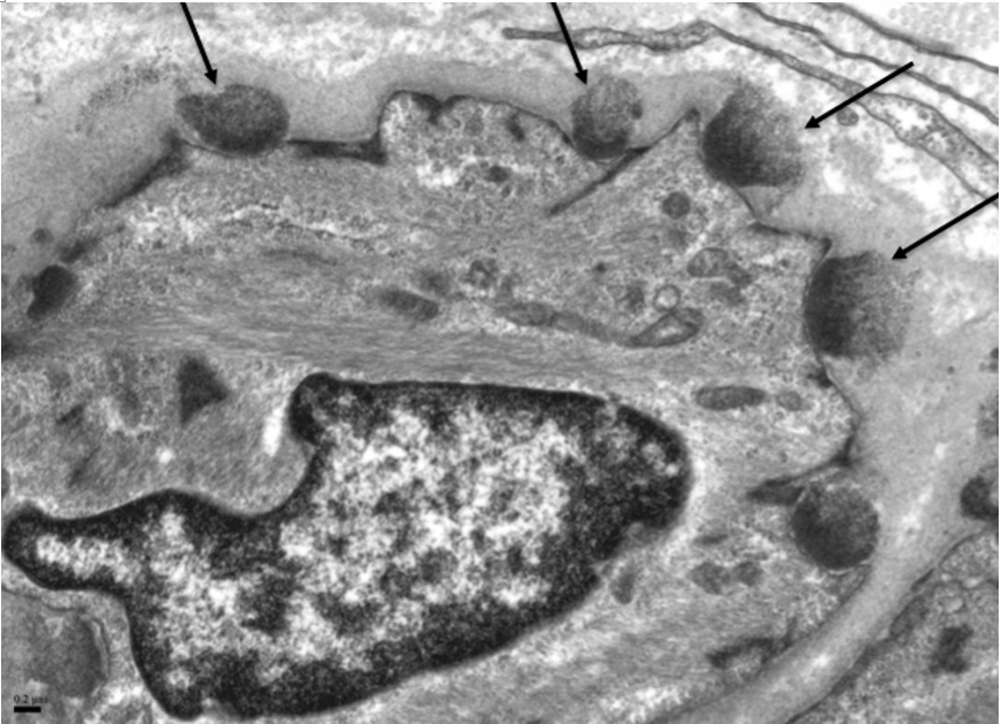
Electron microscopy of skin biopsy shows electron dense granular deposits (arrows) in the basal lamina surrounding vascular smooth muscle cells.

**Fig 3:**
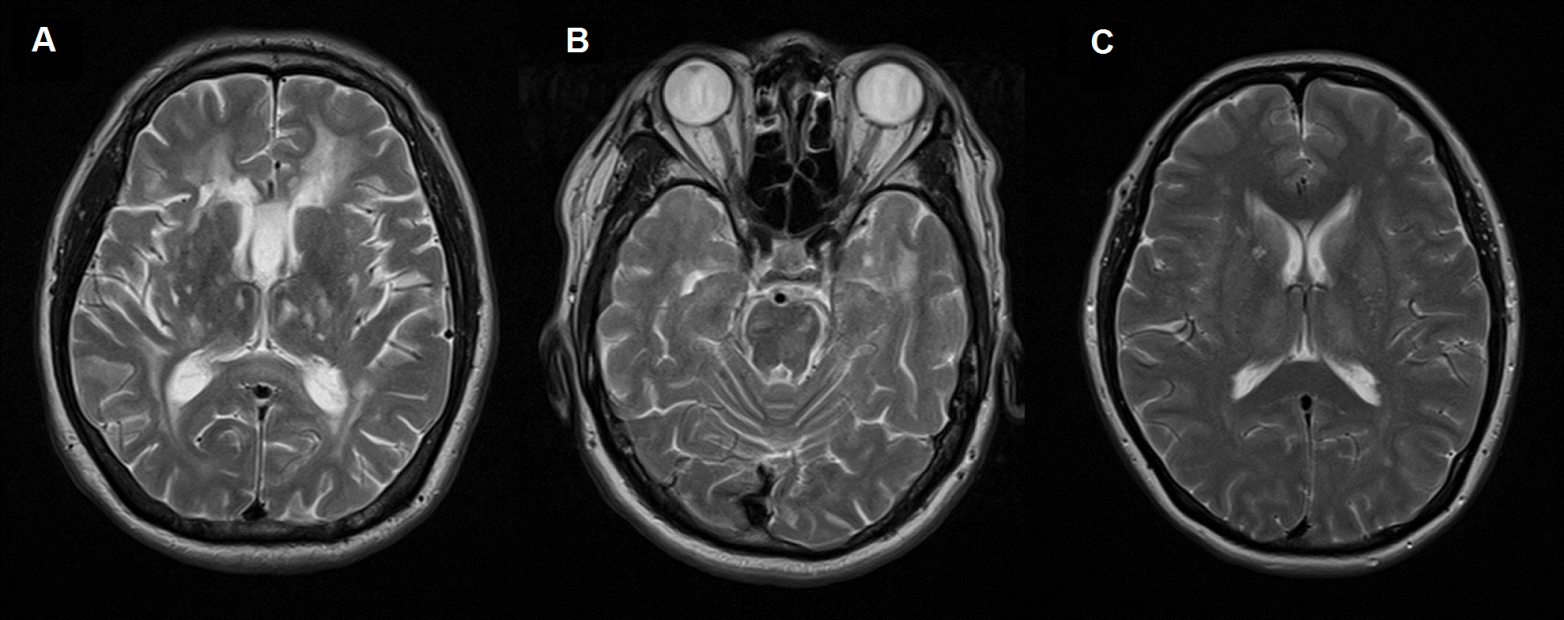
(A): Axial T2W MRI brain of a symptomatic subject (II-9) demonstrates cerebral atrophy. There are confluent hyperintense lesions in the periventricular, subcortical, deep white matter and thalamus, with (B) infra-tentorial and anterior temporal lobe involvement. 3(C): Axial T2W MRI brain of an asymptomatic subject (II-6) demonstrates more discreet deep white matter and periventricular lesions.

The affiliation for the fifth author is incorrect. Ping-Chin Lee is not affiliated with #2 but with #9 Faculty of Science and Natural Resources, University Malaysia Sabah, Jalan UMS, Kota Kinabalu, 88400, Sabah, Malaysia.

In the Funding section, the incorrect author is attributed to the grant. The following grant should be attributed to C-C Ng.: High Impact Research Grant (UMMOHE UM.C/625/1/HIR/MOHE/ CHAN-02 H-50001-A000023), University of Malaya, to C-C Ng.

## References

[1] Lim K-S, Tan A-H, Lim C-S, Chua K-H, Lee P-C, Ramli N, et al (2015) R54C Mutation of *NOTCH3* Gene in the First Rungus Family with CADASIL. PLoS ONE 10(8): e0135470 doi: 10.1371/journal.pone.0135470 26270344 10.1371/journal.pone.0135470 PMC4535948

