# Supplementary material for: R54C Mutation of NOTCH3 Gene in the First Rungus Family with CADASIL
Source: PLoS One. 2015 Aug 13;10(8):e0135470. doi: 10.1371/journal.pone.0135470 (PMC4535948; doi:10.1371/journal.pone.0135470)
Supplement: S1 Table — (DOCX) [file pone.0135470.s001.docx]

**S1 Table.** Primers for PCR and DNA sequencing.

| Exon | PCR product size (bp) | GC content (%) | Primer (5'-3') |
| --- | --- | --- | --- |
| 2 | 149 | 67 | F: TCCTCCACCTTCCTTCAC* |
|  |  |  | R: ACACACAGGGCCCACTGGT* |
| 3-6 | 1164 | 64 | F: TGTGCTGCCCAACCAAGCCA* |
|  |  |  | R: ATCCATGGCTCCCTGCAGAG* |
| 7-10 | 1798 | 60 | F: TTTTGGGCAGAGCAGGAAGA |
|  |  |  | R: ACCCACCTGCCATACAGA* |
|  |  |  | F: CGTTCACACCATAGGGTAGC* |
|  |  |  | R: GTGCCCAGCCCAACACTTT |
| 11-12 | 576 | 63 | F: ATTGGTCCGAGGCCTCACTT* |
|  |  |  | R: ACCCTCGATCTAAGGACCCC |
| 13-16 | 1511 | 61 | F: CTAAGTGGGGTCACGTCGTC |
|  |  |  | F: GGGAGACGGCCATTCTGAAA* |
|  |  |  | R: GACTGTGTTCCCCAGAGCAG |
| 17-21 | 2553 | 61 | F: TCAGACTGGGCTAATGGGGG* |
|  |  |  | R: TCCCTGCTCTCCAAGCAGA |
|  |  |  | F: ATCCTCCCTCCCACTCCTTC |
|  |  |  | R: GTACGTGCATGAGCCCCTTC |
|  |  |  | F: TGTTCCTGTGCCACTCTCCT* |
|  |  |  | R: CAAGAGGAAATGAAGACAGC* |
| 22-23 | 1048 | 60 | F: CATTCCCTCTTGACCACCCC |
|  |  |  | R: CTTTCCACACTGGAGACCCC |
| 24 | 692 | 74 | F: TCTCCTCCTTCCCTCCACTC |
|  |  |  | R: CACGGACAAACAGACTGGGA |

Primers with asterisks were taken from patent US7138234. Primers with underlines were used for both PCR and DNA sequencing. Primers without underlines were used as internal primers for DNA sequencing.

Abbreviations: F, forward primer; R, reverse primer.
