# Supplementary material for: R54C Mutation of NOTCH3 Gene in the First Rungus Family with CADASIL
Source: PLoS One. 2015 Aug 13;10(8):e0135470. doi: 10.1371/journal.pone.0135470 (PMC4535948; doi:10.1371/journal.pone.0135470)
Supplement: S2 Table — (DOCX) [file pone.0135470.s002.docx]

**S2 Table.** PCR working reaction for exons 2 and 24.

| Reagent | Working reaction | |
| --- | --- | --- |
|  | Volume (μl) | Concentration |
| Sterile water | 10.75 | - |
| 5×Q5 reaction buffer | 5 | 1× |
| 5×Q5 high GC enhancer | 5 | 1× |
| dNTPs (10 mM) | 0.5 | 200 μM |
| Forward primer (10 μM) | 1.25 | 0.5 μM |
| Reverse primer (10 μM) | 1.25 | 0.5 μM |
| Q5 hot start DNA polymerase (2 U/μl) | 0.25 | 0.02 U/μl |
| Genomic DNA (50 ng/μl) | 1 | 2 ng/μl |
| Total volume | 25 |  |
