# Supplementary material for: R54C Mutation of NOTCH3 Gene in the First Rungus Family with CADASIL
Source: PLoS One. 2015 Aug 13;10(8):e0135470. doi: 10.1371/journal.pone.0135470 (PMC4535948; doi:10.1371/journal.pone.0135470)
Supplement: S3 Table — (DOCX) [file pone.0135470.s003.docx]

**S3 Table.** PCR working reaction for exons 3-6, 7-10, 11-12, 13-16, 17-21, and 22-23.

| Reagent | Working reaction | |
| --- | --- | --- |
|  | Volume (μl) | Concentration |
| Sterile water | 7 | - |
| 2×phusion flash PCR master mix | 10 | 1× |
| Forward primer (10 μM) | 1 | 0.5 μM |
| Reverse primer (10 μM) | 1 | 0.5 μM |
| Genomic DNA (50 ng/μl) | 1 | 2.5 ng/μl |
| Total volume | 20 |  |
