# Supplementary material for: R54C Mutation of NOTCH3 Gene in the First Rungus Family with CADASIL
Source: PLoS One. 2015 Aug 13;10(8):e0135470. doi: 10.1371/journal.pone.0135470 (PMC4535948; doi:10.1371/journal.pone.0135470)
Supplement: S4 Table — (DOCX) [file pone.0135470.s004.docx]

**S4 Table.** PCR cycling conditions.

| Exon | Initial denaturation | Denaturation | Annealing | Extension | Final extension |
| --- | --- | --- | --- | --- | --- |
|  |  | 30-35 cycles | | |  |
| 2 | 98 ºC; 5 min | 98 ºC; 15 s | 60 ºC; 15 s | 72 ºC; 10 s | 72 ºC; 1 min |
| 3-6 | 98 ºC; 5 min | 98 ºC; 5 s | 68 ºC; 5 s | 72 ºC; 1 min | 72 ºC; 5 min |
| 7-10 | 98 ºC; 5 min | 98 ºC; 10 s | 70 ºC; 10 s | 72 ºC; 1 min | 72 ºC; 5 min |
| 11-12 | 98 ºC; 5 min | 98 ºC; 10 s | 66 ºC; 10 s | 72 ºC; 1 min | 72 ºC; 5 min |
| 13-16 | 98 ºC; 5 min | 98 ºC; 5 s | 66 ºC; 5 s | 72 ºC; 1 min | 72 ºC; 5 min |
| 17-21 | 98 ºC; 5 min | 98 ºC; 10 s | 62 ºC; 10 s | 72 ºC; 1 min | 72 ºC; 5 min |
| 22-23 | 98 ºC; 5 min | 98 ºC; 10 s | 70 ºC; 10 s | 72 ºC; 1 min | 72 ºC; 5 min |
| 24 | 98 ºC; 5 min | 98 ºC; 10 s | 62 ºC; 10 s | 72 ºC; 1 min | 72 ºC; 5 min |
